# Anti-cancer therapeutic benefit of red guava extracts as a potential therapy in combination with doxorubicin or targeted therapy for triple-negative breast cancer cells

**DOI:** 10.7150/ijms.40131

**Published:** 2020-04-06

**Authors:** Hsiao-Chun Liu, Chien-Chuan Chiang, Ching-Hsiang Lin, Chien-Sheng Chen, Chyou-Wei Wei, Shu-Yu Lin, Giou-Teng Yiang, Yung‑Luen Yu

**Affiliations:** 1 Department of Nursing, Taipei Tzu Chi Hospital, Buddhist Tzu Chi Medical Foundation, New Taipei 231, Taiwan; 2 Department of Nutrition, Master Program of Biomedical Nutrition, Hungkuang University, Taichung 433, Taiwan; 3 Graduate Institute of Biomedical Sciences, China Medical University, Taichung 404, Taiwan; 4 enter for Molecular Medicine, China Medical University Hospital, Taichung 404, Taiwan; 5 Department of Emergency Medicine, Taipei Tzu Chi Hospital, Buddhist Tzu Chi Medical Foundation, New Taipei 231, Taiwan; 6 Department of Emergency Medicine, School of Medicine, Tzu Chi University, Hualien 970, Taiwan; 7 Drug Development Center, China Medical University, Taichung 404, Taiwan; 8 Department of Biotechnology, Asia University, Taichung 413, Taiwan

**Keywords:** Red guava, Triple-negative breast cancer, Apoptosis, Necrosis

## Abstract

Guava extracts purified from leaf and bark have many bio-active molecules with anti-cancer activities. In addition, lycopene-rich extracts obtained from red guava fruit can induce apoptosis in estrogen receptor-positive breast cancers. Triple-negative breast cancer (TNBC) lacks estrogen receptors, progesterone receptors and human epidermal growth factor receptor 2 (HER2) and, therefore, hormone therapy and targeted therapy are not used in the clinic. The purpose of this study was to determine whether red guava fruit extracts can affect the proliferation of TNBC cells. In this study, cell viability was determined by using the MTT assay. Apoptosis and necrosis were analyzed using flow cytometry. Cleaved caspase-3 and PARP were analyzed by western blotting. We found that red guava extracts can, through caspase-3 activation and PARP cleavage signaling, induce apoptotic and necrotic death in TNBC cells. Our results thus show the therapeutic benefit of red guava extracts as a potential cancer treatment for TNBC in combination with doxorubicin or targeted therapy.

## Introduction

Many studies have demonstrated that guava extracts have anti-inflammatory, anti‑microbial, anti‑oxidative and anti-cancer activities 1-3. These functions of guava extracts are related to numerous bio-active molecules such as beta-caryophyllene oxide, vitamins, tannins, phenolic compounds, flavonoids and triterpenoid acids 2,4-6. Previous studies have demonstrated that guava extracts can inhibit cell growth in various cancers such as lung cancer, colorectal carcinoma, myeloid leukemia, squamous cell carcinoma, myeloma, cervical cancer and gastric cancer 3,7,8. However, the mechanisms induced by guava extracts that result in anti-cancer activities remain unknown.

Breast cancer is the second major cause of death among cancers that are generally diagnosed in women 9,10. Breast cancer commonly has invasive characteristics and a high rate of metastasis, which result in high morbidity and mortality 11,12. Currently, chemotherapy, hormone therapy and targeted therapy are the most common breast cancer treatments 13,14. About 15-20% of breast cancer occurrences are triple-negative breast cancer (TNBC) 15. TNBC lacks estrogen receptors (ERs), progesterone receptors (PRs) and epidermal growth factor receptor 2 (HER2), and, therefore, chemotherapy, hormone therapy and targeted therapy are not useful for TNBC treatment 16-18. Thus the development of a method for treating TNBC is important.

Some studies have suggested that guava extracts, most of which were purified from guava leaves or guava bark, are potential materials for breast cancer treatment 5,19,20. Guava extracts are suggested to have estrogen-like activity, and, therefore, guava extracts are useful for treating ER-positive breast cancer cells, such as MCF-7 cells 19. Although aqueous extracts of guava fruit may not display effective anti-cancer activity 21, lycopene-rich extracts from red guava fruit can induce apoptosis-like cell death among ER-positive breast cancer cells 22. Thus different guava species have different bioactive components that result in different activities. However, whether lycopene or other bioactive molecules from red guava fruit have anti-cancer activities on TNBC cells remains to be studied.

Doxorubicin, a DNA topoisomerase II inhibitor, has been used as a chemical therapy for cancers, including breast cancer 23,24. However, doxorubicin is not effective for TNBC treatment. Other agents may be used to enhance doxorubicin-induced anti-cancer activity on TNBC 25. Tarceva (Erlotinib) and Iressa (Gefitinib), both of which are EGFR inhibitors, are used as targeted therapy for breast and other cancers 26,27. Because EGFR is frequently overexpressed in TNBC, but up to now EGFR inhibitors are not useful drugs for TNBC treatment. To enhance the anti-cancer activity of tarceva and iressa on TNBC, combination therapy is required 28,29. In this study, we show that extracts from red guava fruit can promote anti-cancer activities in iressa-, tarceva- and doxorubicin-treated TNBC.

## Materials and methods

### Materials

Red guavas were kindly provided by a farmer, Lin Chao Hsiung (A Fong guava farm, Houbi Dist., Tainan City 731, Taiwan). Doxorubicin, Erlotinib (Tarceva; Roche, Basel, Germany) and Gefitinib (Iressa; Astra Zeneca, London, England) were dissolved in dimethyl sulfoxide (DMSO) and the concentrations of drugs were referred to previous studies 15,25,30. Doxorubicin was obtained from China Medical University Hospital. Antibodies against caspase-3 (cat no. 9662), caspase-8 (cat no. 9746), caspase-9 (cat no. 9502), PARP (cat no. 9542) and cleaved caspase-3 (cat no. 9664) were obtained from Cell Signaling Technology (Beverly, MA, USA). The MTT assay kit and antibodies against α-tubulin (cat no. T607) and β-actin (cat no. A2228) were purchased from Sigma (St. Louis, MO, USA). Amicon Ultra Filters (≤30 kDa; No. UFC900324) were purchased from Millipore (Billerica, MA, USA). Fluorescein isothiocyanate-labeled annexin V (annexin V-FITC), propidium iodide (PI) and Western Lightning Plus-ECL enhanced chemiluminescent substrate were purchased from Perkin Elmer (EU, USA).

### Cell lines and cell culture

The human mammary epithelial cell line MCF-10A and human TNBC cell lines MDA-MB-231 and MDA-MB-468 were purchased from American Type Culture Collection. These cells were maintained in a humidified atmosphere containing 5% CO_2_ at 37ºC and cultured in Dulbecco's modified Eagle's medium plus F12 (DMEM/F12 = 1:1) supplemented with 10% fetal bovine serum, penicillin (100 U OR IU OR UI/mL) and streptomycin (100 µg/mL; all from Sigma). In addition, insulin (10 µg/mL; Sigma) was used for MCF-10A cells.

### Guava extract preparation

Guava extracts were prepared as described 1. Briefly, extracts from the mature stage of the red guava fruit were used in this study. The fruits were cut and seed sections removed before the flesh was ground with a pure juice machine (NationalMJ-C85N) to prepare a crude juice without extra water. The crude juice was centrifuged at 4,000 × *g* (BECKMAN COULTER Allegra X-15®) for 30 min, and the supernatant was collected (total extract). The lower molecular weight (**≤** 30 kDa) extracts (LMW extracts) were obtained from total guava extracts centrifuged at 4ºC, 4000 × *g* for 30 min in an Amicon Ultra Filter. Finally, total extracts and LMW extracts were filtered with a 0.22-μm filter and stored at -80ºC until use. The protein concentration of total extracts and LMW extracts was ~1.8 g/ml.

### Cell viability assay

Cell viability was determined with the MTT assay kit. Cells were cultured in 96-well plates (6 × 10^3^ cells/well). After the indicated treatments, control and experimental cells were incubated for 3 h at 37ºC with components from the MTT assay kit. The absorbance of the reactive product was measured at 570 nm by using a Multiskan™ FC Microplate Photometer (Molecular Devices, Sunnyvale, CA, USA). Cell viability is indicated as a percentage corresponding to (*A*_570_ experimental group)/(*A*_570_ control group) × 100.

### Cell cycle analysis

Cell cycle analysis was conducted using fluorescence‑activated cell sorting. Cells from control and experimental groups were washed with phosphate-buffered saline (PBS) and fixed with 70% ethanol at 4ºC for 1 h. The fixed cells were washed with PBS for 5 min and then treated with 1 ml PI solution containing 50 μg/ml PI, 100 μg/ml RNase A and 0.1% Triton X‑100 for 30 min at 37ºC. The cells were then washed with PBS and analyzed by flow cytometry (Partec CyFlow® SL; SysmexPartec GmbH, Görlitz, Germany). The resulting data were analyzed with CellQuest software (Becton-Dickinson).

### Apoptosis and necrosis assay

Apoptosis and necrosis were determined by annexin V-FITC/PI staining. Briefly, control and experimental cells were collected and washed with cold PBS buffer. Cells were resuspended in 100 µL Annexin V/PI buffer and then treated with 5 µL of annexin V**-**FITC and 1 µL of PI for 15 min in the dark (at room temperature). Viable, apoptotic (annexin V^+^) and necrotic (PI^+^/ annexin V^-^) cells were analyzed by flow cytometry and quantified with CellQuest software.

### Western blotting

The cells were treated with RIPA buffer (EMD Millipore, Billerica, MA, USA) and centrifuged (16,000 × *g* at 4ºC) for 20 min, and then cellular proteins were collected from the supernatant layer. Protein concentrations were determined with a Bradford protein assay kit (Bio-Rad, Hercules, CA, USA). Equal quantities (20 μg) of protein were loaded onto an SDS-polyacrylamide gel and separated by electrophoresis. The proteins were transferred from the gel to PVDF membranes (EMD Millipore). The membranes were blocked with 5% non-fat milk solution for 2 h at room temperature and were then washed with TBST buffer three times. The membranes were further incubated with primary antibodies at 4°C overnight. After being washed with TBST three times, the membranes were incubated with secondary antibodies for 1 h at room temperature. Finally, the membranes were treated with Western Lightning® Plus-ECL (Perkin Elmer, EU, USA), and immunolabeled proteins were observed using a Luminescence Image Analysis System (LAS-4000, FUJIFILM Electronic Materials Taiwan Co., Ltd., Tainan).

### Statistical analysis

Data were collected from three independent experiments and are indicated as the mean ± SD. Means were analyzed with the t-test method by using Microsoft Excel (http://microsoft-excel-2010.updatestar.com/zh-tw). A P-value < 0.05 was considered statistically significant. *P < 0.05, **P < 0.01.

## Results

### Extracts from red guava fruit decrease cell viability in TNBC cells

**Figure 1:**
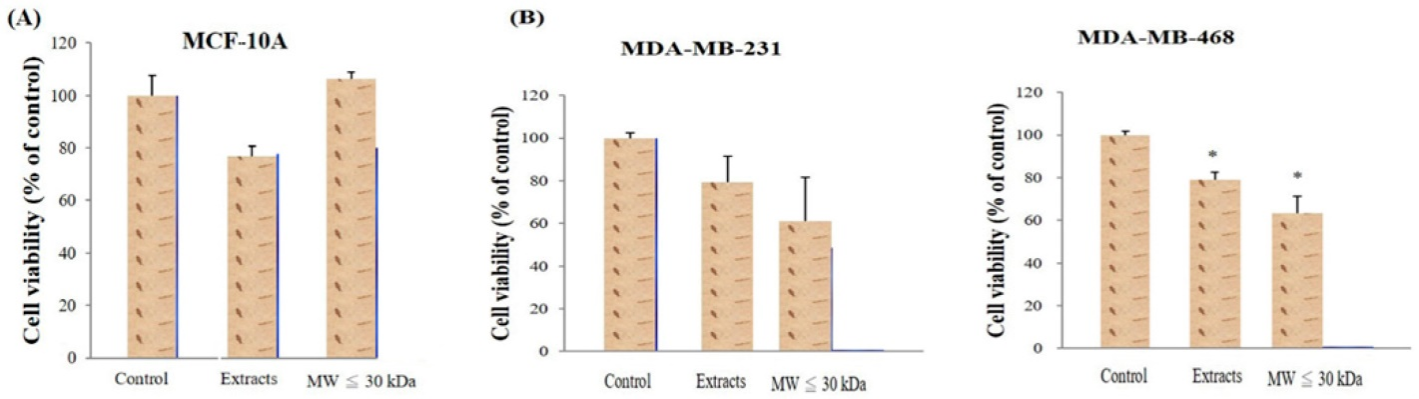
** Determination of red guava extract effects on cell viability**. (A) Human mammary epithelial cells (MCF-10A) were treated with no guava extract (control), 10% total extract (extracts) or 10% LMW extracts (MW ≤ 30kDa) for 72h at 37ºC. (B) TNBC cells (MDA-MB-231 and MDA-MB-468) were treated with no guava extract, 10% total extract or 10% LMW extract as in A. The 72-h cell viability was measured by MTT analysis. Values are expressed as the mean ± standard deviation from three independent experiments. *P < 0.05.

Two extract types from red guava fruit were analyzed: total extracts and smaller molecular weight (<30 kDa) extracts (LMW extracts). The two extract types were first incubated with normal breast cells (MCF-10A) and no extract type decreased the viability of MCF-10A cells (Fig. 1A). Total extracts and LMW extracts were also used to treat TNBC cells (MDA-MB-231 and MDA-MB-468) under the same conditions. Both extracts decrease the viability of the TNBC cells (Fig. 1B). Moreover, comparted with control group, the viability of MDA-MB-468 was significantly difference in guava extract-treated group with a P-value < 0.05.

### Extracts from red guava fruit induce cell cytotoxicity in MDA-MB-231 and MDA-MB-468 cells via different cell death pathways

**Figure 2:**
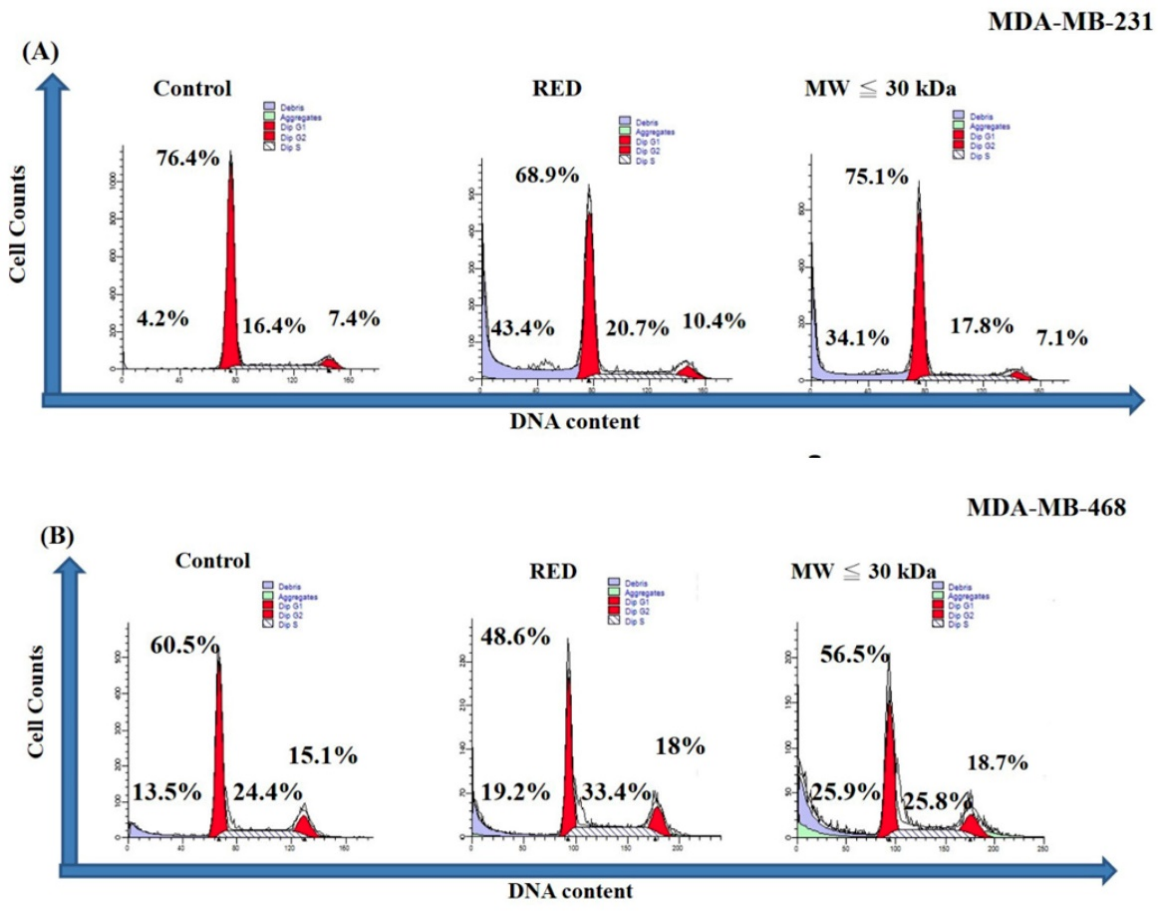
** Cell cycle analysis of TNBC cells treated with red guava extracts.** (A) MDA-MB231 and (B) MDA-MB-468 cells were treated with **no** guava extract (control), 10% total extract (RED) or 10% LMW extract (MW ≤ 30kDa). The cells were fixed with ethanol and stained with PI. The cell cycle after extract exposure for 72 h was analyzed by flow cytometry.

**Figure 3:**
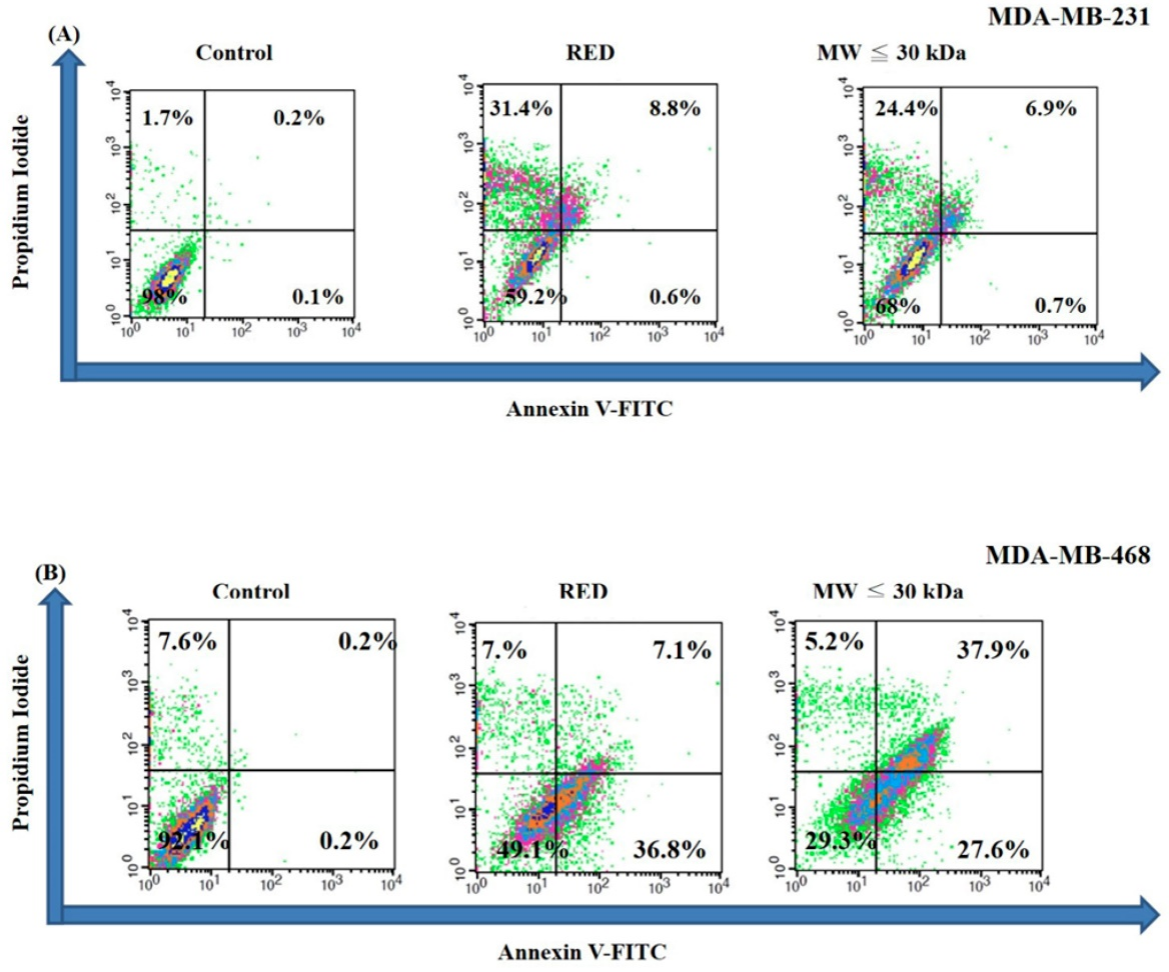
** Apoptotic and necrotic cell death analysis in TNBC cells treated with red guava extracts**. (A) MDA-MB231 and (B) MDA-MB-468 cells were treated with no guava extract (control), 10% total extract (RED) or 10% LMW extracts (MW ≤ 30kDa). After a treatment period of 72 h, the cells were stained with annexin V-FITC and PI and analyzed using flow cytometry.

A cell cycle analysis was carried out on TNBC cells treated with total extracts and LMW extracts for 72h. As shown in Figure 2A, the sub-G1 percentages of MDA-MB-231 cells were 4.2%, 43.4% and 34.1% in the control, total extract and LMW extract groups, respectively. On the other hand, the sub-G1 percentage of MDA-MB-468 was increased with guava extracts treatment with sub-G1 percentages of 13.5%, 19.2% and 25.9% in the control, total extract and LMW extract groups, respectively (Fig. 2B). Thus both the total extracts and LMW extracts from red guava fruit increased the sub-G1 percentage of TNBC cells. That is, red guava extracts were able to induce cell cytotoxicity in TNBC cells. Cell death is generally classified as apoptotic cell death or necrotic cell death. We used an annexin V/PI assay to determine the occurrence of apoptotic and necrotic cell death in these cells. As shown in Figure 3A, MDA-MB-231 cells that were treated with total extract (31.4% necrosis and 9.4% apoptosis) or LMW extract (24.4% necrosis and 7.6% apoptosis) had higher necrosis and apoptosis percentages as compared with control cells (1.7% necrosis and 0.3% apoptosis). Thus total extract and LMW extract induced a higher percentage of dead cells as a result of the necrotic death pathway rather than the apoptotic death pathway in MDA-MB-231cells. In contrast, as shown in Figure 3B, MDA-MB-468 cells that were treated with total extract (7.0% necrosis and 43.9% apoptosis) or LMW extract (5.2% necrosis and 65.5% apoptosis) had only a higher apoptosis percentage as compared with control cells (7.6% necrosis and 0.4% apoptosis). Thus total extract and LMW extract induced apoptosis in MDA-MB-468 cells.

### Extracts from red guava fruit induce cell death in MDA-MB-231 and MDA-MB-468 cells via PARP cleavage and caspase-3 activation

**Figure 4:**
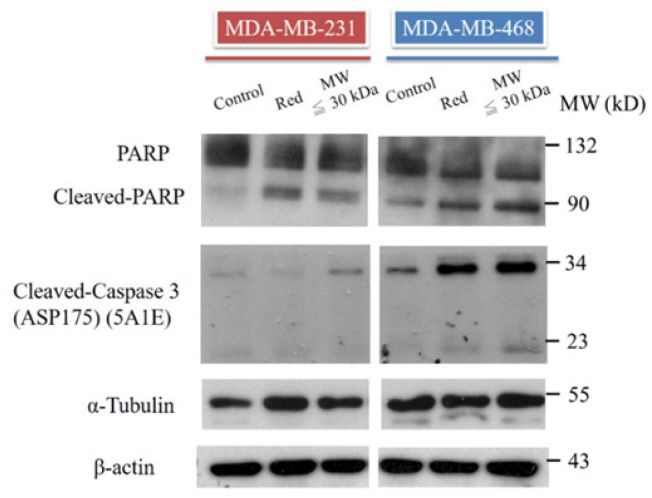
** PARP, cleaved PARP and cleaved caspase-3 analysis in TNBC cells treated with red guava extracts**. The protein levels of PARP, cleaved PARP and cleaved caspase-3 were assayed by western blotting. Cells were treated with **no** guava extract (control), 10% total extract (RED) or 10% LMW extract (MW ≤ 30kDa). Proteins were analyzed after 72 h of treatment. The protein levels of tubulin and actin were used as loading controls.

PARP and caspase-3 activity were assessed in TNBC cells treated with total extract and LMW extract. Based on western blotting, cleavage of PARP was increased in MDA-MB-231 and MDA-MB-468 cells treated with both extract types (Fig. 4). Caspase-3 is the upstream signal of PARP, and, therefore, caspase-3 activation was also analyzed. Cleavage of caspase-3 was increased in the total extract and LMW extract groups (Fig. 4). These data suggested that extracts from red guava fruit activate the caspase-3/PARP signaling pathway to induce cytotoxicity in TNBC cells.

### Extracts from red guava fruit promote chemotherapy-induced and targeted therapy-induced anti-cancer activities

**Figure 5:**
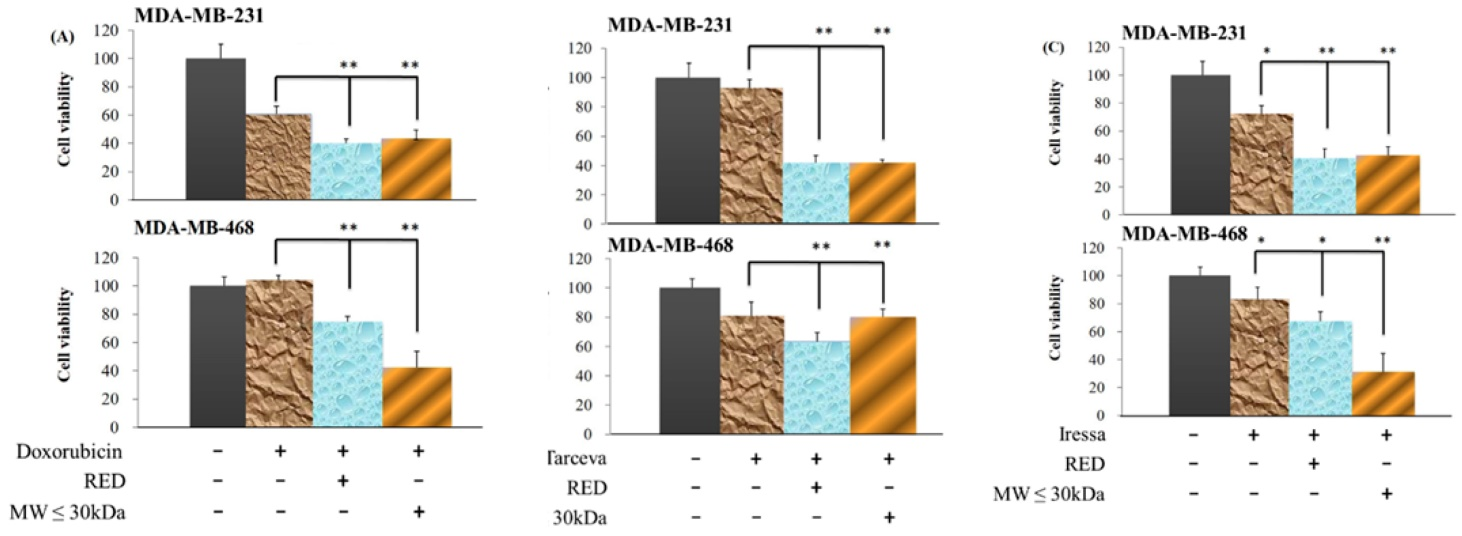
** Cell viability of TNBC cells treated with guava extracts plus clinical anti-cancer drugs.** (A) Cells were treated with 0 nM doxorubicin (Control; column 1), 17 nM doxorubicin (column 2), 17 nM doxorubicin plus 10% total extracts (RED; column 3) or 17 nM doxorubicin plus 10% LMW extracts (MW ≤ 30kDa; column 4). (B) Cells were treated with 0 μM tarceva (column 1), 1 μM tarceva (column 2), 1 μM tarceva plus 10% total extracts (column 3) or 1 μM tarceva plus 10% LMW extracts (column 4). (C) Cells were treated with 0 μM iressa (column 1), 1 μM iressa (column 2), 1 μM iressa plus 10% total extracts (column 3) or 1 μM iressa plus 10% LMW extracts (column 4). The 72-h cell viability was measured by the MTT method. Values are expressed as the mean ± standard deviation from three independent experiments. *P < 0.05, **P < 0.01.

TNBCs lack the ER, PR and HER2, and, therefore, many clinical therapies are not effective for the treatment of TNBCs. Doxorubicin, a DNA topoisomerase II inhibitor, is used as a chemotherapy for many cancers including TNBCs. To confirm that doxorubicin is broadly effective against TNBC cells, we treated MDA-MB-231 and MDA-MB-468 cells with doxorubicin for 72h. Doxorubicin inhibited MDA-MB-231 cells; however, doxorubicin was not toxic to MDA-MB-468 cells (Fig. 5A). When extracts from red guava fruit (total extracts group and LMW extracts) were combined with doxorubicin treatment, the anti-cancer activities were significantly increased in MDA-MB-231 and MDA-MB-468 cells (Fig. 5A). Tarceva and Iressa both of which are EGFR inhibitors, are used as clinical targeted therapies for breast cancer. In our study, neither Tarceva nor Iressa effectively inhibited MDA-MB-231 or MDA-MB-468 cells (Fig. 5B and C). However, extracts from red guava fruit (total extracts group and LMW extracts) when combined with Tarceva or Iressa significantly increased their anti-cancer activities in MDA-MB-231 and MDA-MB-468 cells (Fig. 5B and C). Taken together, our findings suggested that extracts from red guava fruit could promote chemotherapy- and targeted therapy-induced anti-cancer activities.

## Discussion

Many studies have demonstrated that guava extracts have anti-cancer activities on various cancers including lung cancer, colorectal carcinoma, myeloid leukemia, squamous cell carcinoma, myeloma, cervical cancer, gastric cancer and breast cancer 3,7,8,19. However, whether guava extracts can inhibit TNBC growth had not been examined. Here we have shown that extracts from red guava fruit can induce cell death in TNBC cells. Most studies have shown that guava extracts induce large amounts of apoptotic death in various cancer cells 7,22,31. Our study, in contrast, found that red guava extracts induce apoptotic and necrotic cell death in TNBC. Specifically, red guava extracts induced mainly necrotic cell death in MDA-MB-231 cells (more metastasis ability than MDA-MB-468), but they induced apoptotic cell death in MDA-MB-468 cells. Therefore, we suggest that red guava extract can lead to distinct activation of cell death pathways in different types of TNBC cells may dependent on their malignancy.

A previous study showed that extracts purified from guava fruit do not have anti-cancer activity in breast cancer cells 21. However, extracts obtained from lycopene-rich red guava fruit can inhibit ER-positive breast cancer growth 22. Here we also found that extracts purified from red guava fruit can inhibit TNBC growth, with LMW extracts and total extracts having similar cytotoxic effects on these cells. Lycopene is <30 kDa, and thus the LMW extracts in this study likely contained lycopene molecules. Previous studies have demonstrated that lycopene has anti-cancer activities 32-34. Based on the experimental results from our study and previous studies, we believe that lycopene is one of the important molecules in red guava fruit with respect to anti-cancer effects on TNBC cells.

Combination treatment of etoposide and doxorubicin can enhance cytotoxicity in TNBCs through the TRAIL-DR5 axis 25. In addition, TRAIL signals can increase NF-kB activity 35, and guava extracts can regulate NF-kB activity 3. Our data showed that red guava fruit extracts can promote doxorubicin-induced cytotoxicity in TNBC cells, but whether red guava extracts enhance this cytotoxicity via TRAIL/NF-kB signaling remains to be studied.

In support of another possible signaling pathway, combination treatment with EGFR inhibitors and PI3K/AKT inhibitors has a synergistic effect on TNBC 29. Recently, a study reported that guava extracts can activate PI3K and AKT 36. In our analysis, red guava extracts enhanced the cytotoxicity of EGFR inhibitors (tarceva and iressa) in TNBC cells. Therefore, we suggest that the PI3K/AKT pathway may play an important role in TNBC treatment.

Taken together, our study demonstrated that red guava extracts display anti-cancer activities in TNBC cells through apoptotic or necrotic cell death pathways and that red guava extracts promote doxorubicin-, tarceva- and iressa-induced cytotoxicity in TNBC cells.
